# Patterns of pollen and nectar foraging specialization by bumblebees over multiple timescales using RFID

**DOI:** 10.1038/srep42448

**Published:** 2017-02-09

**Authors:** Avery L. Russell, Sarah J. Morrison, Eleni H. Moschonas, Daniel R. Papaj

**Affiliations:** 1 Graduate Interdisciplinary Program in Entomology and Insect Science, University of Arizona, Tucson, AZ, 85721, USA; 2 Lunar and Planetary Laboratory, University of Arizona, Tucson, AZ, 85721, USA; 3 Department of Ecology and Evolutionary Biology, University of Arizona, Tucson, AZ, 85721, USA

## Abstract

The ecological success of social insects is frequently ascribed to improvements in task performance due to division of labour amongst workers. While much research has focused on improvements associated with lifetime task specialization, members of colonies can specialize on a given task over shorter time periods. Eusocial bees in particular must collect pollen and nectar rewards to survive, but most workers appear to mix collection of both rewards over their lifetimes. We asked whether bumblebees specialize over timescales shorter than their lifetime. We also explored factors that govern such patterns, and asked whether reward specialists made more foraging bouts than generalists. In particular, we described antennal morphology and size of all foragers in a single colony and related these factors to each forager’s complete foraging history, obtained using radio frequency identification (RFID). Only a small proportion of foragers were lifetime specialists; nevertheless, >50% of foragers specialized daily on a given reward. Contrary to expectations, daily and lifetime reward specialists were not better foragers (being neither larger nor making more bouts); larger bees with more antennal olfactory sensilla made more bouts, but were not more specialized. We discuss causes and functions of short and long-term patterns of specialization for bumblebee colonies.

Task specialization is a hallmark of insect societies^1^,^2^. Eusocial bees, some of our most important pollinators, must engage in a variety of tasks over their lifetime, including nest construction, brood care, and foraging from flowers to feed themselves and their nest mates. Specialization on different foraging tasks in particular has been well studied (e.g., refs 3 and 4). Because switching between tasks can incur temporal, cognitive, and/or energetic costs, specialization is thought to maximize task efficiency^3^,^4^,^5^,^6^. For instance, bees often have to learn new nectar collection routines each time they shift to a new plant species^3^,^7^. Cognitive costs associated with learning and recalling such collection routines are thought to make it advantageous for foraging bees to specialize in the short term on a given plant species^3^,^4^,^5^,^6^,^7^,^8^. In addition, individuals can vary in their task performance as a result of fixed physiological or morphological differences; for example, honeybees vary in their sucrose sensitivity^4^,^9^. In fact, intrinsic differences among foragers are thought to explain lifetime patterns of specialization on the collection of nectar, water, and pollen in foraging honeybees^9^,^10^. These and other studies suggest that patterns of foraging task specialization by individual worker bees might differ when we examine the short term (e.g., hours or days) versus the long-term (e.g., lifetime). Yet almost no research has examined specialization by the same foragers over different timescales.

Patterns of foraging specialization over different timescales have important implications for how individuals and, for social bee species, colonies manage the collection of multiple floral rewards. The two most common floral rewards collected by bees are pollen and nectar^11^,^12^. Flowering plants offer pollen and nectar in all combinations and qualities. For instance, the flowers of some plant species offer pollen and nectar, only pollen, only nectar, or even vary the availability of one or the other reward over the floral lifecycle or as a result of prior collection. Thus a forager might not always be able to collect pollen and nectar on a single floral visit (e.g., refs 13 and 14). Further, collecting both rewards during a single foraging bout may not be efficient if, for instance, the bee has to travel far to collect both rewards. In addition, colony needs vary over lifetime. Bees rely on nectar as their primary source of carbohydrates and pollen as their primary source of protein and lipids^15^,^16^. While worker bees primarily consume nectar, larvae consume prodigious amounts of pollen^16^,^17^. Changes in the amount of brood (e.g., ref. 18) and in storage of one or the other food type (e.g., refs 12, 17 and 19) can therefore change the relative need for each resource. Given the dynamic nature of the resource environment and nutrient demands of individuals and the colony, patterns of individual reward specialization (i.e., the ratio of pollen to nectar collection bouts a given forager makes) might be expected to change over time.

Apart from a relative dearth of information about patterns of reward specialization, we know even less about the mechanistic factors that govern reward specialization^20^. However, one body of literature ascribes variation in reward specialization to intrinsic individual differences in behaviour and sensory properties. According to the well-supported response threshold model (RTM), reward specialization arises as a result of interindividual variation in sensitivity to cues that stimulate nectar or pollen foraging (so called ‘response thresholds’^9^,^10^). In honeybees, reward specialization and corresponding variation in response thresholds are associated, at least in part, with variation in sensory structures^10^. Specifically, differences in the number of antennal olfactory sensilla (i.e., sensilla placodea) correspond to differences in reward specialization, independent of variation in body size^10^. In particular, honeybee workers with low response thresholds (high sensitivity) typically forage for pollen, whereas workers with high response thresholds (low sensitivity) typically forage for nectar^9^,^10^.

In this study, we assessed patterns of individual reward specialization in bumblebees, and the possible role of sensory morphology in driving these patterns. Relative to other eusocial bee species, bumblebees (*Bombus*) exhibit considerable within-colony variation in body size (i.e., alloethism). Large body size is thought to be associated with greater foraging efficiency; larger bees of a given species have better sensory systems for detecting and assessing flowers (e.g., allometric scaling results in bigger eyes and more antennal olfactory sensilla), can carry more nutrients before returning to the nest (e.g., bigger storage organs), and can forage further afield^21^,^22^,^23^,^24^. Yet any association between body size and reward specialization remains ambiguous. We predict that larger bumblebee workers, which should have low response thresholds due to greater numbers of antennal olfactory sensilla, will be more specialized on pollen. Alternatively, we might expect that specialists on either reward would be larger and thus more effective foragers; olfactory sensilla number might instead relate more strongly to the degree to which a forager is specialized on a given reward.

To test these hypotheses, we compiled a complete lifetime record of pollen and nectar foraging activity for every bumblebee forager (*Bombus impatiens*) in a single colony and related these patterns to the sensory morphology and size of each forager. We used radio frequency identification (RFID) technology to track bees as they moved between foraging chambers that presented either only pollen or only nectar from artificial flowers. We measured antennal sensory morphology (sensilla placodea number or ‘pore plates’) and other morphological attributes (forewing length, head width, antennal length, and proboscis length) which might be associated with different patterns of reward specialization and foraging effort. This is the first study to our knowledge that examines patterns of reward specialization at multiple timescales and that connects them to forager size and sensory morphology.

## Methods

### Bees

**Figure 1:**
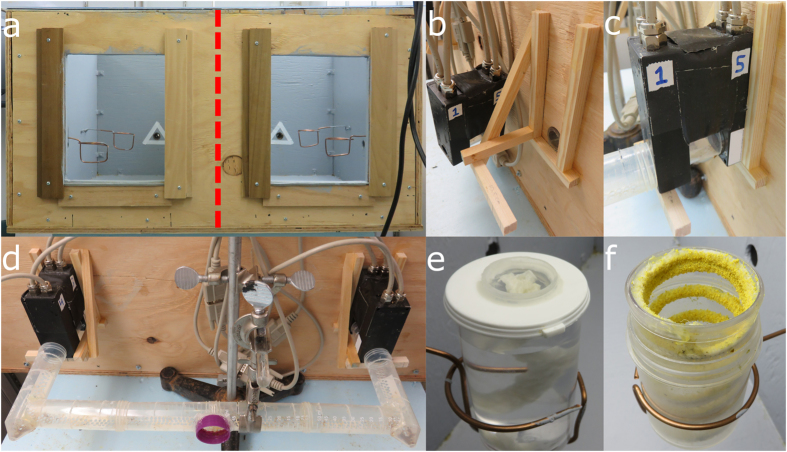
Elements of the experimental foraging setup. (**a**) Forward view of the foraging arena with each foraging chamber in view. Four of the 6 custom-built copper feeder-holders can be seen. The red-dashed line indicates the location of the plywood wall separating the arena into two foraging chambers. The white triangles surround the chamber entrances. (**b**) Paired RFID reader holder and chamber entrance. (**c**) Paired RFID readers mounted on holder, joined to tunnel. (**d**) Paired RFID readers and forking tunnel setup. Purple ring attaches to a tunnel (not shown) that in turn connects to the colony box. (**e**) Nectar feeder. (**f**) Pollen feeder.

We used a single colony of *Bombus impatiens* in a study conducted between September 2013 and January 2014. The 4-week old colony was purchased from Koppert Biological Systems (Howell, MI, USA) and had 12 workers when it arrived. The colony was allowed to acclimate until new foragers began to eclose 23 days later, whereupon the colony was attached to the experimental foraging setup described below. During acclimation, bees were trained to forage for pollen and nectar in a foraging arena (LxWxH 58 × 73 × 40 cm) that provided *ad libitum* honeybee-collected pollen (Koppert Biological Systems, MI, USA) and 1 M sucrose solution. A single feeder dispensed sucrose solution via braided cotton wicks (6 inch Braided Cotton Rolls, Richmond Dental) that extended into 40 dram vials through perforations made in the white plastic lids (BioQuip Products, Inc.) (Fig. 1e). Pollen was presented from a single custom-made feeder^25^ consisting of chenille fibres (white to the human eye), glued to the inside walls of 40 dram vials (BioQuip Products, Inc., USA)(Fig. 1f). Besides the natural scent and colour of the sucrose solution and pollen, neither feeder was scented or coloured. The experimental setup was maintained at 26° Celsius.

After the colony was attached to the experimental foraging setup bees that died were frozen at −18 °C and stored in individually labelled BEEM capsules (Ted Pella, Inc.). Two dead bees were too damaged for use in subsequent morphological analyses. We did not check the parasite load of bees, which might have affected bee behaviour (e.g., ref. 26).

### Experimental foraging setup

An arena constructed of plywood (LxWxH 58 × 73 × 40 cm) was divided into two equal-sized foraging chambers (each LxWxH 58 × 36 × 40 cm) via a removable plywood wall (Fig. 1a). The floor and sides of the arena were painted grey. The arena had a clear acrylic ceiling and was lit from above by 40 W 60 Hz fluorescent lights (Lithonia Lighting). The colony was kept dark while lights in the arenas were set to a 12:12 light:dark cycle.

One entrance hole was drilled through the outer wall of each foraging chamber (Fig. 1b). The colony box (accessible via a single hole) was joined to the foraging chambers by a branching tunnel with ends attached to each chamber (Fig. 1d). Two RFID readers (MAJA reader module 4.2, Microsensys GmbH, GE) were mounted in sequence on custom holders at the entrance of each foraging chamber (Fig. 1c). As bees moved through the paired readers, the identity of the chamber and direction of movement (into versus out of the chamber) was automatically recorded.

One chamber provided *ad libitum* honeybee-collected pollen and the other provided 1 M sucrose solution. Three feeders dispensed sucrose solution and three feeders dispensed pollen. Feeders were of the design previously described. Feeders were suspended 17 cm from the floor of the chamber (measured from the top of the feeder) by way of custom-built holders (Fig. 1a). This design presented a more realistic foraging situation by preventing those bees that could not (or would not) fly from foraging.

### Tagging procedure

Unlabelled bees that left the colony box were tagged with 1.5 × 1.0 × 0.5 mm RFID transponders (mic3-tag 64 RO, Microsensys GmbH, GE). Each transponder (‘tag’) weighed 2.5 mg; approximately 2.6% of the weight of a bee (mean bee weight in mg for this colony ± SE: 95.5 ± 4.7), 4.9% of a nectar load^27^, or 15% of a pollen load (A. Russell unpub. data). To find foragers that had not yet been tagged, we checked the foraging arena and tunnels every 15–60 minutes, 8 hours each day, 6 days a week (Monday-Saturday). Unlabelled foragers were briefly trapped in a part of the tunnel that had a removable bottom and thereby captured in 40 dram vials (BioQuip Products, Inc.). Foragers were immobilized in a Queen Marking Tool (The London Bee Company Ltd, Middlesex, UK) and a single tag glued to the dorsum of their thoraxes with a small amount of superglue. Shortly (<1 minute) thereafter bees were individually placed into vials with pollen scent to calm them (this eliminated aggression by other bees to the newly-labelled bee; A. Russell pers. obs.), then transferred back into the colony after approximately 2 minutes.

### Morphological measures

**Figure 2:**
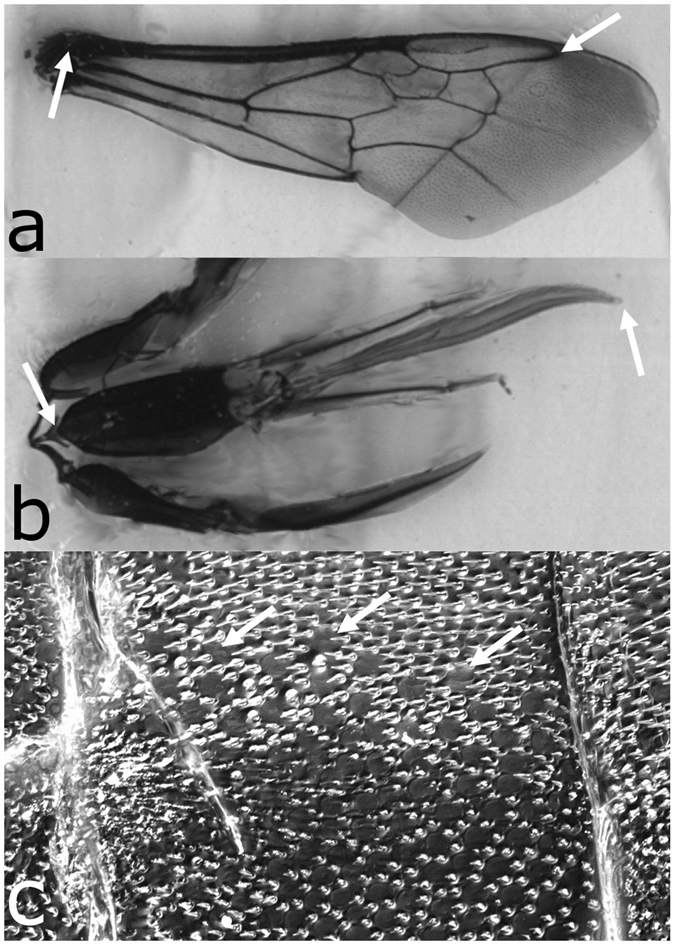
Representative *Bombus impatiens* forewing and proboscis scanned at 2400dpi and portion of an antenna’s nail polish cast. (**a**) Right forewing and (**b**) proboscis. White arrows indicate start and end points for length measures made in ImageJ. (**c**) Nail polish cast of the 7^th^ antennal segment showing the boundary between the zone with pore plates (bottom) and the zone with only hair plates (top). White arrows indicate several pore plates at the boundary.

On each bee we measured head width, forewing length, proboscis length, antennal flagellum length, and the 7^th^ antennal flagellomere’s width and length, and number of pore plates. Measurements were done as follows. The head of each bee was first removed and photographed in frontal view at 1.2X using a digital camera with a 5.2 mega pixel resolution (DCM500 Microscope CMOS Camera, Microscope Cameras), affixed within the ocular of a stereoscope. Next, the proboscis (proximal base of postmentum to distal tip of labellum), antennae (at the scape), and forewings (without damaging the basal articular sclerites) were removed. The antennae were flat-mounted and photographed at 2X via the digital camera setup. The proboscis and forewings were sealed between two clear transparencies (Staples Transparency Copy Film, Staples, Inc.) using mounting medium and then scanned at 2400dpi (Epson Perfection 3490 Photo, Epson America, Inc.). ImageJ (National Institutes of Health, Bethesda, MD, http://imagej.nih.gov/ij/) and a micrometre were used to make and calibrate all measures, respectively. Head widths were measured at their widest point. Forewing length was measured from the distal edge of the marginal cell (the distal-most leading vein at the point where R1 and R meet; Fig. 2a), to the base of the radius, at which point it meets the sclerite. Proboscis length was measured from proximal base of prementum to distal tip of labellum (Fig. 2b). We measured the centre-line length of the antennal flagellum. In addition, the width and length of the 7^th^ antennal segment were measured and the surface area of the structure calculated, assuming a cylinder.

To count sensilla, antennae were coated in a thin layer of clear nail polish (Hard as Nails, Sally Hansen) and suspended in air for 3 minutes to dry. Antennal casts were cut open lengthwise with a scalpel, making sure to cut only through the area of the antennae that exhibited hair plates (sensilla trichodea) and not pore plates (sensilla placodea) (Fig. 2c). Casts were peeled off and mounted flat between a slide and coverslip so that we could examine the complete surface of the antenna in one focal plane. We focused on the pore plates (sensilla placodea) because of their established function in olfaction^28^ and because they differ between honeybee pollen and nectar foraging specialists^10^. Because our methodology did not produce intact casts for all antennal segments, we made drawings of the casts of antennal segment 7 (segments 8–10 were sometimes damaged) and all its pore plates at 10X magnification, via a camera lucida mounted to a compound microscope (Leitz Laborlux). We focused on this distal segment because pore plates are more numerous on more distal antennal segments^29^ and differences amongst bees might therefore be more readily quantified. The pore plate density on the 7^th^ segment was calculated using our ImageJ measurements.

There were small differences between forewings in a pair (<3%) and between antennae in a pair (<7%), but there was no overall bias across bees for one particular side for either forewings or antennae (Supplementary Materials); thus when left and right measures were available we always used the larger of the two.

### Calculating the amount of brood

The colony was photographed through the Plexiglas cover from above each day. We used a custom-built stand to hold the camera at constant height and location. Using ImageJ we traced the perimeter of the colony (the wax comb) and the perimeter of the brood cells (completely closed cells). ImageJ’s ‘area’ function was used to determine the percent of the colony’s surface composed of brood cells. Because young colonies tend to grow outward, on the surface of the nest box, rather than upward, as older colonies tend to do (A. Russell pers. obs.), this measurement of brood is likely relatively accurate.

### RFID data processing software

Custom-written MATLAB R2013b software was used to process RFID data and extract foraging related parameters. Throughout this manuscript we define a ‘foraging bout’ (or ‘bout’) as a bee entering a foraging chamber and staying within it for more than 60 seconds, but less than 30 minutes (see Supplementary Materials for detailed information on scoring foraging bouts). For a subset of the data (days 1–5), 98.6% of visits within the foraging chamber lasted less than 30 minutes (mean bout duration in minutes ± SE: 6.98 ± 0.15). Additionally, we visually confirmed for 1 hour of observations for each of 5 consecutive days that 100% of bees that spent less than 60 seconds in the flight arena did not forage (they entered and then left the arena rapidly; mean length of all visits in seconds for days 1–5 = 22.4 seconds) and that approximately 80% of bees in the foraging chamber were actually collecting sucrose (proboscis extended and abdomen pumping on the sucrose feeder) or pollen (bee packing pollen into its pollen baskets) at any given time. Although foragers were not prevented from visiting both the nectar and pollen chamber before returning to the colony, all of the observed foragers returned to the colony after foraging from a single chamber.

### Data analyses

All data were analysed using R v.3.2.0 30.

We tested for a significant association between lifetime degree of reward specialization and lifetime foraging period for foragers using a linear model (LM). Possible values range from 0.5 to 1: values closer to 0.5 belong to bees that collected both rewards equally frequently; values closer to 1 belong to bees that were specialized on a given reward (either pollen or nectar). The LM was specified using the lm() function in R. ‘Degree of reward specialization’ was specified as the independent variable and ‘lifetime foraging period’ was specified as the response variable. We log transformed the independent variable and thereby normalized the residuals.

To determine how foragers switched between foraging for pollen versus nectar across bouts while controlling for any bias in their reward preference, we applied a modified Jacob’s Constancy Index^31^, calculated as 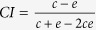, where ‘c’ was the proportion of transitions between the same reward (i.e., the proportion of sequences made to one reward following an immediately prior bout made to that same reward) and where ‘e’ was the proportion of transitions between the same reward given the expected proportion (the overall frequency of nectar bouts over the lifetime of the forager). Possible values range from −1 to 1: bees that were more inconstant (systematically alternated between rewards) have values closer to −1; bees that made random transitions between rewards have values closer to 0; bees that were more constant (i.e., foraged in runs for one or the other reward) have values closer to 1. We used a one-way Wilcoxon signed rank test to determine whether bees on average foraged in runs.

The 14 bees that showed complete constancy (CI = 1) or complete inconstancy (CI = −1) made very few bouts over their lifetime (18 or fewer bouts, as compared to a mean ± SE of 115.6 ± 15.3 bouts over forager lifetime; *N* = 98 foragers), raising the possibility that these CI values were a result of small sample size. We therefore discarded all bees that had made 18 or fewer bouts from this analysis (22 total bees). Mean Jacob’s CI dropped as a result (mean ± SE: before, 0.18 ± 0.04; after, 0.11 ± 0.03).

We ran a Chi-square (χ^2^) test via the chisq.test() function in R to analyse whether lifetime reward specialists made proportionally fewer bouts. Likewise, we ran a paired *t*-test via the t.test() function in R to analyse whether daily reward specialists made proportionally fewer daily bouts.

We used separate linear models, after model selection (see Supplementary Materials), to determine whether morphological characteristics were associated with behavioural patterns.

We report results of two sets of linear models (LMs). For one set we report effects of ‘pore plate number’ on lifetime behaviour (‘total days foraged’, ‘lifetime bouts’, ‘lifetime nectar bouts’, ‘lifetime pollen bouts’, ‘lifetime nectar preference’). For the other set we report effects of ‘forewing length’ on mean daily behaviour (‘mean daily bouts’, ‘mean daily nectar bouts’, ‘mean daily pollen bouts’). We apply a conservative Bonferroni correction for these analyses (α-value = 0.006). We discarded 22 bees that had zero values in at least one category (otherwise we encountered errors).

In addition, we used separate LMs to determine effects of colony age on brood and foraging characteristics, applying a Bonferroni correction (α-value = 0.017). Colony age was specified as the independent variable for each LM. Daily ‘percent brood’, ‘colony reward preference’, ‘foraging force size’, ‘mean bouts per forager’, percentage of daily ‘reward specialists’ (>90% of bouts per day for either food type), percentage of daily ‘nectar specialists’, or percentage of daily ‘pollen specialists’ were specified as the response variable. For the LM examining the effect of colony age on the size of the active foraging workforce (foraging force size) we used an *F*-test via the R function anova() to test whether a quadratic model was better than a linear model. The quadratic model was a much better fit (*F*_1,40_ = 74.272, *P* < 0.0001, *N* = 43 days) and we therefore only report the results of the quadratic model. For the LM examining the effect of colony age on daily percent brood, we discarded 10 days for which photos of brood analysis were not available (due to file corruption); these missing days were distributed approximately evenly.

We also used an LM to determine whether forager lifetime nectar preference was associated with mean daily measures of foraging performance. Log-transformed measures of foraging performance (‘daily nectar bouts’ and ‘daily pollen bouts’) were specified as fixed factors and forager lifetime nectar preference was specified as the dependent variable. To examine the possible significance of an interaction between daily nectar bouts and daily pollen bouts, results were first examined using type III sums of squares via the ‘Anova()’ function in the car package^32^,^33^. Because the interaction was not statistically significant, we report results with a Type II ANOVA via the ‘Anova()’. In a separate LM, we used the offset() function in R to change the null hypothesis of the linear regression to a slope of 1.We discarded 14 bees that had zero values in at least one category (otherwise we encountered errors).

We used another LM to determine the effect of when a forager joined the foraging workforce on the mean daily number of bouts made by the forager. ‘Date of joining workforce’ was specified as the independent variable and ‘mean daily bouts’ was specified as the response variable. Both variables were log-transformed, thereby normalizing the residuals.

We used separate linear mixed effects models (LMM) to determine the effect of forager age on the number of bouts made for each forager per day and on their daily degree of reward specialization. ‘Forager age’ was specified as the independent variable and also included as a repeated measures factor within the random factor ‘BeeID’. Models were specified via the lmer() function in the lmerTest package^34^. We report the overall result via type II Wald chi-square (χ2) tests with Anova().

To determine whether morphological characteristics (‘forewing length’, ‘head width’, ‘proboscis length’, ‘pore plate number’, ‘antennal length’, ‘7^th^ antennal segment width’, ‘7^th^ antennal segment length’, and ‘pore plate density’) were correlated with one another via Pearson’s *r* we used the R function cor(). Correlations between lifetime behavioural measures (‘total days foraged’, ‘lifetime bouts’, ‘lifetime nectar bouts’, ‘lifetime pollen bouts’, ‘lifetime nectar preference’) were assessed in the same way. Correlations are reported in the Supplementary Materials.

## Results

We obtained a complete foraging record for the growth phase of the colony (43 days; from eclosion of the first new workers to eclosion of the first reproductives, marking the end of worker replacement and colony growth). Of 111 RFID-tagged bees, 103 made at least one foraging bout after being tagged. The colony made 11507 foraging bouts (mean per day: 267.6 bouts); 7801 for nectar (mean bout length in minutes ± SE: 7.62 ± 0.06) and 3706 for pollen (mean bout length in minutes ± SE: 5.64 ± 0.09).

### Most foragers were reward generalists over lifetime, but specialists in the short term

**Figure 3:**
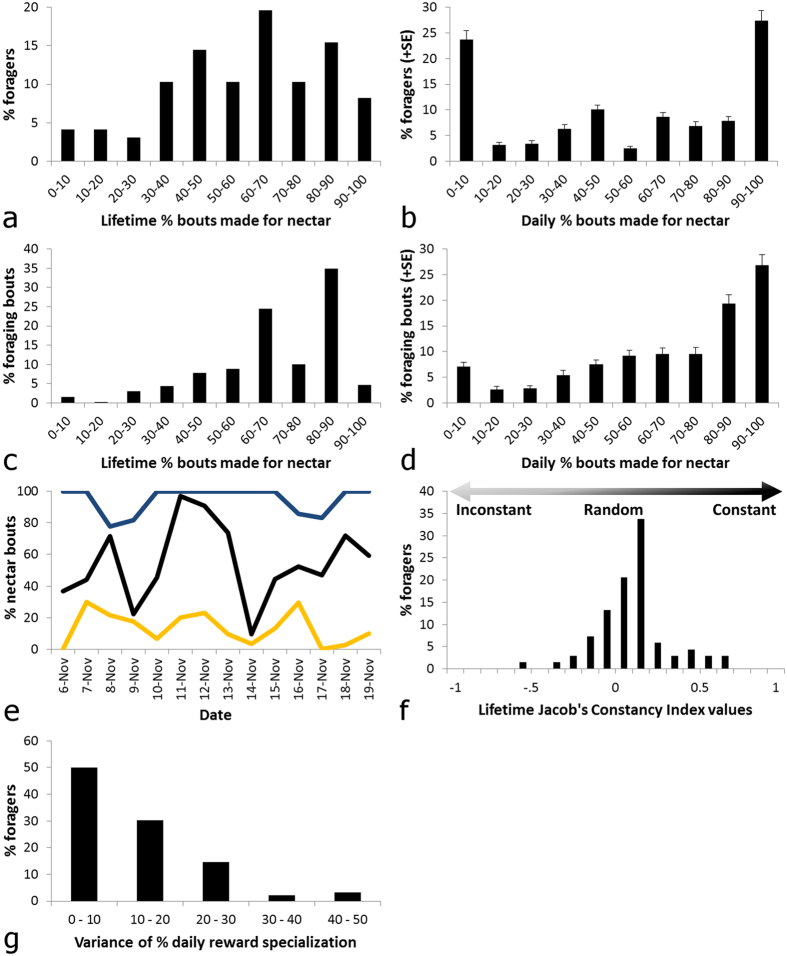
Patterns of reward specialization and of foraging effort for bees with a given degree of specialization, over both short and long timescales. Percentage of foragers with a given (**a**) lifetime or (**b**) mean daily nectar foraging preference (+SE). Percentage of (**c**) colony lifetime or (**d**) mean daily foraging bouts made by foragers with a given (**c**) lifetime or (**d**) mean daily nectar foraging preference (+SE). Bin width = 10%; range = 0.0–100.0% nectar foraging preference; *N* = 97 bees and 43 days. (**e**) A 2-week foraging period for exemplars of the three major types of reward specialists: a bee that specialized on nectar collection over its lifetime (top trace - blue line); a bee that specialized on pollen collection over its lifetime (bottom trace - yellow line); and a bee that specialized on either food type over the short-term (middle trace - black line). (**f**) Jacob’s Constancy Index (CI) for forager lifetime: bees that were more inconstant (systematically alternated between rewards) have values closer to −1; bees that made random transitions between rewards have values closer to 0; bees that foraged in runs for one or the other reward have values closer to 1; bin width = 0.1; *N* = 68 bees. (**g**) Variance of percentage daily reward specialization; bin width = 10; *N* = 97 bees.

There was a continuous distribution of lifetime reward preference among foragers (Fig. 3a). Only a minority (12.4%) of foragers could be classified as long-term reward specialists (making > 90% of their foraging bouts for a single type of reward over their lifetime^1^,^20^) on either food type. Degree of lifetime reward specialization, ranging from 1 (completely specialized on a given reward) to 0.5 (collecting both rewards equally frequently) was not significantly associated with an individual’s lifetime foraging period (LM: *F*_1,96_ = 2.92, *P* = 0.091, *R*^2^ = 0.019).

Conversely, short-term reward specialization was common: on average, 51.1% of a day’s foragers made > 90% of their bouts for a single type of reward (Fig. 3b; see Fig. 3e top and bottom traces for examples). Many of these foragers switched between specializing on either pollen or nectar over a day or more, thereby mixing rewards over their lifetimes (see Fig. 3e middle trace for an example). Additionally, bees exhibited substantial flexibility in their daily degree of reward specialization (Fig. 3g). Despite mixing rewards, most bees foraged in runs for one or the other reward: the mean Jacob’s Constancy Index (CI) value for all foragers over lifetime was significantly greater than zero (Fig. 3f; one-way Wilcoxon signed rank test: *V* = 1858, *P* < 0.0001, *N* = 68 bees; mean Jacob’s CI ± SE: 0.11 ± 0.03). Likewise, most foragers (73.5% of 68 foragers) had a CI > 0.

### A small number of foragers contributed disproportionately to foraging effort

**Figure 4:**
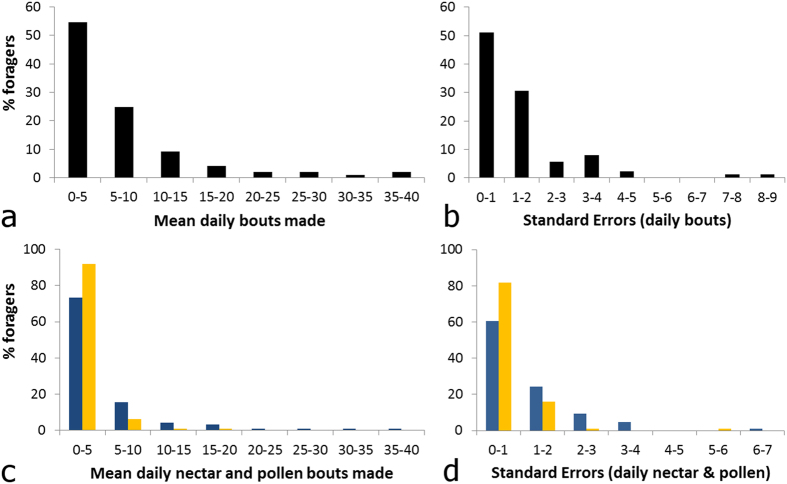
Percentage of foragers that make a given daily mean number of foraging bouts and standard error (S.E.) around the means. (**a,b**) Nectar and pollen bouts summed (**c,d**) nectar (blue bars) and pollen (yellow bars) bouts displayed separately. Bin width for means = 5 bouts; range: all bouts, 1.0–38.4 bouts per day; nectar bouts, 0.0–35.8; pollen bouts, 0.0–15.85. Bin width for variances = 1 bout; range: all bouts, 0.2–8.7; nectar bouts, 0.1–6.9; pollen bouts, 0.1–5.6. *N* = 97 bees and 43 days.

Mean daily foraging effort, estimated as mean number of daily foraging bouts, varied nearly 40-fold among foragers; foraging effort formed a continuous, though highly skewed distribution (Fig. 4a). In fact, half of the colony’s mean number of daily foraging bouts were performed by a minority (17.3%) of foragers (mean daily bouts ± SE: 20.7 ± 2.2, *N* = 17 bees). Conversely, when foragers were ranked by activity level, the bottom 50% made just 16.7% of the colony’s daily foraging bouts (mean daily bouts ± SE: 2.4 ± 0.1, *N* = 49 bees). Variation in mean daily nectar foraging effort was more substantial than variation in mean daily pollen foraging effort (Fig. 4c).

Importantly, short (daily) and long-term (lifetime) reward specialists (foragers that made > 90% of their bouts for a single type of reward for the given duration) contributed little to foraging effort: long-term reward specialists (12.4% of 97 total foragers) made just 6.3% of all foraging bouts made by the colony (Fig. 3a,c); short-term specialists (51.1% of 33.1 ± 1.4 foragers on average) likewise made only 33.9% of mean daily foraging bouts (Fig. 3b,d). Proportionally, daily reward specialists made significantly fewer foraging bouts each day (paired *t*-test: null expectation of equivalence of % of bouts made by reward specialists and % of foragers each day that were reward specialists, *t*_42_ = 10.991, *P* < 0.0001, *N* = 43 days). Although not significant, lifetime reward specialists also tended to make fewer foraging bouts (χ^2^-test: null expectation that % bouts made by reward specialists = % foragers that were reward specialists, χ^2^ = 1.99, *P* = 0.158).

### More active foragers preferred to collect nectar over lifetime

**Figure 5:**
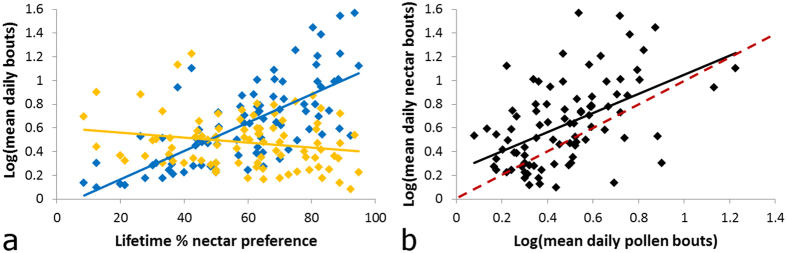
Log of the mean daily number of foraging bouts made by foragers with a given lifetime nectar collection preference. (**a**) Lifetime nectar foraging preference plotted against nectar (blue points) and pollen (yellow points) bouts, displayed separately, (**b**) nectar bouts plotted against pollen bouts; slope of 1 indicated by red dashed line. *N* = 89 bees. Plots with trend lines indicate a statistically significant relationship (p < 0.05) according to a Type II ANOVA and an LM, respectively.

Forager preference to collect nectar was strongly associated with mean daily number of bouts made for nectar and pollen (Fig. 5a; LM overall effect: *F*_3,85_ = 1429, *P* < 0.0001, *R*^2^ = 0.98, *N* = 89 bees). Specifically, increasing preference to collect nectar was strongly positively associated with more daily nectar bouts and, to a lesser degree, negatively associated with more daily pollen bouts (Fig. 5a; Type II ANOVA: log(daily nectar bouts) effect: *F*_1,85_ = 4062.1, *P* < 0.0001; log(daily pollen bouts) effect: *F*_1,85_ = 1847.8, *P* < 0.0001). There was no significant interaction between mean daily nectar bouts and mean daily pollen bouts (Type II ANOVA: daily bouts:daily nectar bouts effect: *F*_1,85_ = 0.2817, *P* = 0.597). Further, foragers making the most mean daily bouts tended to be less strongly specialized on nectar collection (Fig. 5b; Type II ANOVA: slope < 1 for LM of log(daily nectar bout) on log(daily pollen bout): *F*_1,87_ = 76.722, *P* < 0.0001).

### Effects of colony age on reward preference, specialization, and amount of brood

**Figure 6:**
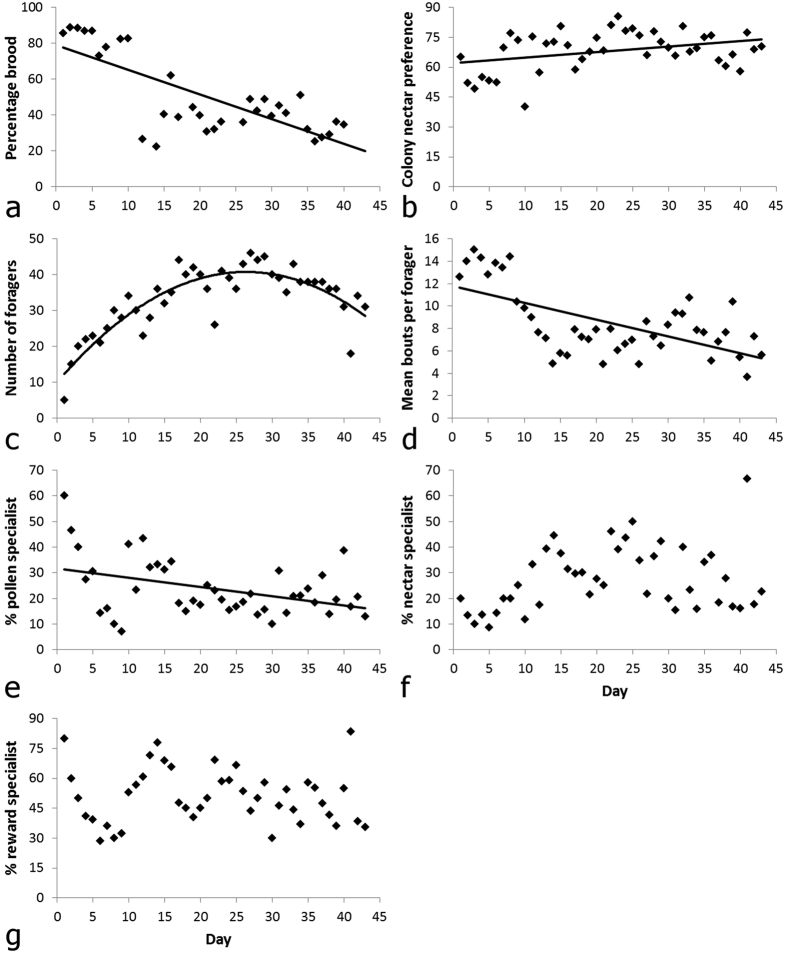
Effects of colony age on its foraging behaviour and on its reproduction. (**a**) Percent of the colony surface composed of brood cells, (**b**) colony nectar collection preference, (**c**) number of active foragers, (**d**) mean number of bouts per bee, (**e**) percentage of bees that specialized on nectar collection, (**f**) percentage of bees that specialized on pollen collection, and (**g**) percentage of bees that specialized on either food type, for each day of the experiment. *N* = 89 bees. *N* = 33 days for (**a–d**) and 43 days for (**e–g**). Plots with trend lines indicate a statistically significant relationship (p < 0.05) according to LMs; although the analysis for (**c**) was performed via a quadratic model, the quadratic fit for the untransformed data is shown here.

Amount of brood, colony reward preference, and the size of the active foraging workforce all changed significantly over the lifetime of the colony (*N* = 43 days; Fig. 6a–d). As the colony aged, the amount of brood (percent colony surface composed of brood cells) declined significantly and the colony shifted to foraging significantly more for nectar relative to pollen (Fig. 6a–b; LM: age effect on: percent brood, *F*_1,31_ = 42.82, *P* < 0.0001, *R*^2^ = 0.580; colony reward preference, *F*_1,41_ = 5.838, *P* < 0.014, *R*^*2*^ = 0.139; Bonferroni correction α-value = 0.017). Furthermore, as the colony aged, the active foraging workforce increased significantly in size and the mean daily foraging bouts per forager decreased significantly (Fig. 6c–d; LM: age effect on: foraging force size, *F*_1,41_ = 59.85, *P* < 0.0001, *R*^*2*^ = 0.737; mean bouts per forager, *F*_1,41_ = 25.89, *P* < 0.0001, *R*^*2*^ = 0.387; Bonferroni correction α-value = 0.017). Although a forager’s mean daily foraging rate did not change significantly with forager age (LMM: forager age effect, χ^2^ = 0.708, *P* = 0.401, *N* = 103 bees), foragers that joined the workforce later in the colony’s life made significantly fewer mean daily bouts than foragers that joined the workforce earlier (LM: date of joining workforce effect on mean daily bouts, *F*_1,96_ = 4.29, *P* = 0.041, *R*^2^ = 0.043).

Patterns of reward specialization also changed significantly over the lifetime of the colony (*N* = 43 days; Fig. 6e–g). Specifically, as the colony aged the percentage of bees that specialized daily on pollen significantly decreased, while there was a trend for the percentage of bees that specialized daily on nectar to increase (Fig. 6e–f; LM: age effect on: % pollen specialists, *F*_1,41_ = 8.124, *P* < 0.007, *R*^*2*^ = 0.165; % nectar specialists, *F*_1,41_ = 3.555, *P* = 0.066, *R*^*2*^ = 0.080; Bonferroni correction α-value = 0.017). There was no significant correlation between percentage of daily reward specialists and colony age (>90% of bouts per day for either food type) (Fig. 6g; LM: age effect on: % reward specialists, *F*_1,41_ = 0.207, *P* = 0.651, *R*^*2*^ = 0.005; Bonferroni correction α-value = 0.017). A forager’s daily degree of reward specialization decreased as it aged (LMM: forager age effect, χ^2^ = 6.42, *P* < 0.012, *N* = 103 bees).

### Bees with more pore plates foraged more for pollen

**Figure 7:**
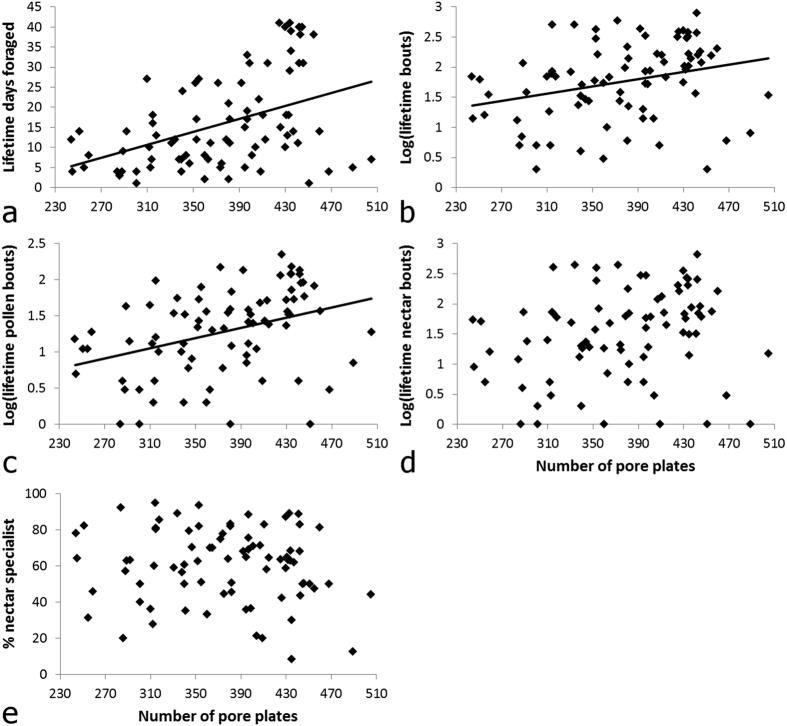
The relationship between forager antennal sensory morphology and lifetime foraging patterns. Lifetime number of (**a**) active foraging days, (**b**) nectar and pollen bouts summed, (**c,d**) pollen and nectar bouts displayed separately, and (**e**) the lifetime preference of foragers to collect nectar. The number of pore plates is reported for the 7^th^ antennal segment of each forager. *N* = 81 bees. Plots with trend lines indicate a statistically significant relationship (p < 0.05) according to LMs.

Bees with more antennal pore plates foraged for more days and made more pollen bouts over their lifetime (Fig. 7a–c; LM: pore plate number effect on: total days foraged, *F*_1,79_ = 17.52, *P* < 0.0001, *R*^2^ = 0.182; log(lifetime bouts), *F*_1,79_ = 7.18, *P* < 0.009, *R*^2^ = 0.083; log(lifetime pollen bouts), *F*_1,79_ = 12.4, *P* < 0.0008, *R*^2^ = 0.136; Bonferroni correction α-value = 0.006; pore plate number varied by a factor of 2.09 across all foragers). There was no significant association between pore plate number and a forager’s lifetime number of nectar bouts or the degree to which a forager preferred to collect nectar over its lifetime (Fig. 7d–e; LM: pore plate number effect on: log(lifetime nectar bouts), *F*_1,79_ = 3.97, *P* = 0.05, *R*^2^ = 0.048; lifetime nectar preference, *F*_1,79_ = 0.518, *P* = 0.474, *R*^2^ = 0.007; Bonferroni correction α-value = 0.006).

Forewing length (a proxy for body size) was not associated with mean daily number of bouts (for pollen, nectar, or both) (LM: forewing length effect on: log(daily pollen bouts), *F*_1,79_ = 6.54, *P* < 0.013, *R*^2^ = 0.076; log(daily nectar bouts), *F*_1,79_ = 0.807, *P* = 0.372, *R*^2^ = 0.010; log(daily bouts), *F*_1,79_ = 2.341, *P* = 0.130, *R*^2^ = 0.029; Bonferroni correction α-value = 0.006; *N* = 81 bees; forewing length varied by a factor of 1.83 across all foragers). In other words, mean daily activity level was not related to forager morphology.

## Discussion

Previous research has characterized foraging specialization on the two dominant floral rewards, pollen and nectar, at a particular stage in the colony’s lifespan, for a subset of foragers, and/or at particular times of day (e.g., for honeybees, stingless bees, and bumblebees^20^,^32^,^35^,^36^,^37^). These data snapshots, while informative, offer limited insight into daily and lifetime patterns of reward use for the individual and colony. For bumblebees in particular, the limited evidence to date indicates that individual workers specialize weakly on reward type over their lifetimes (e.g., refs 1, 20 and 38). However, how bees mix collection of pollen and nectar over shorter timescales and how these patterns may change over the life of the colony has so far been unclear. One of the reasons we do not have this information is that it has been logistically difficult to obtain. With the aid of RFID technology, we obtained a complete description of patterns of reward specialization for all bumblebee foragers in the colony during its growth phase. Logistics dictated the use of a single colony, due to the labour intensive nature of obtaining a colony lifetime record of foraging and forager morphology. Future work should examine between-colony variation, as forager behaviour can differ greatly between colonies (e.g., refs 39 and 40). We therefore advise caution when drawing definitive conclusions from our work.

We found that patterns of specialization differed greatly depending on the timescale over which specialization was assessed. Only a small percentage of foragers specialized on one or the other food type over their lifetimes (making > 90% of their bouts to a single reward), in accordance with what has been described previously in the literature (e.g., refs 1 and 20). Yet while most foragers were reward generalists over their lifetimes, greater than half of all foragers specialized on one or the other food type on a given day.

Why might the colony benefit from having individuals specialize on a given food type over short timescales, while having them switch specialization periodically over their lifetime? Foragers might specialize over short timescales for multiple reasons. For instance, costs of switching might be particularly high over short timescales. Costs associated with switching between tasks are thought to reduce task performance, thus encouraging a greater degree of task specialization by workers^5^,^41^. Alternatively, individuals may not be able to track changes in colony requirements precisely enough to justify more frequent switching. For instance, if it takes substantial time for a given forager to ascertain shifts in colony demand, frequent switching is likely to realize little or no benefit in relation to its costs.

There is reason to believe that individuals cannot instantaneously track changes in colony requirements. Colony food stores and cues released by the brood drive collection of pollen and nectar (e.g., refs 12, 18 and 19). Actively foraging workers likely interact sparingly with colony stores and brood. Conversely, workers that are not actively foraging (e.g., during night-time hours) likely interact frequently with colony stores and brood. Consequently, an active forager might respond little to changes in colony demand over the foraging day. Instead, the forager would rely on an internal motivational state set by prior knowledge of the colony’s needs, obtained while that worker was not actively foraging. Upon again attending primarily to tasks within the nest, the forager’s internal motivational state would be reset. Such a mechanism of setting and resetting forager motivational state could explain why most bees specialize over short timescales when costs of acquiring information on colony resource demand are high, but switch specializations over longer timescales when costs are periodically lower.

Why then do foragers switch specializations periodically? By having foragers be flexible in their specialization, the colony might rapidly and efficiently meet shifting resource demands. For instance, pollen and nectar availability change rapidly over several days or weeks as flowers open, are depleted by visitors, and senesce and as new resource patches become available or decline (see ref. 42). Although we controlled the foraging environment, the colony itself was a changing environment. Colonies have been shown to exhibit strong day-to-day variation in demand for one or the other food type (e.g., refs 12, 18 and 19). Accordingly, we found that when brood - which requires copious pollen to develop - was abundant, pollen was the food type of choice for short-term specialists. When brood declined, bees were more likely to specialize on nectar collection in the short term.

If a pattern of individual specialization with periodic switching leads to gains in colony foraging performance we might expect those gains to be associated with higher individual foraging performance^5^. Indeed, data snapshots of prior research suggests that at least some putative nectar and pollen specialists in a colony forage at a higher rate or contribute more to colony foraging effort over a short duration (e.g., refs 1, 41 and 43). However, the complete description of both reward specialization and foraging effort provided by our RFID system allowed us to assess this metric of foraging performance for all foragers in a colony across multiple timescales. Interestingly, lifetime specialists made proportionally fewer foraging bouts than lifetime generalists. This result may not be particularly surprising, because lifetime generalists are also putatively daily reward specialists (specializing on either reward on any given day). However, we were surprised to find that daily reward specialists made proportionally fewer foraging bouts than generalists at the same timescale. One possible explanation is that daily generalists may have been specializing at a different timescale (e.g., shorter or longer than a day) than examined in this study.

Additionally, daily reward specialists might have been better foragers had they been relatively larger. Larger bees are thought to be better foragers because they are able to carry more resources per bout and are able to locate and learn about resources more quickly due to their disproportionately larger sensory organs (e.g. refs 21, 22, 23, 24). However, we found no relationship between degree of lifetime reward specialization and body size. The lack of a relationship should not come as a surprise: while some foragers were consistent across days in the degree and type of reward on which they specialized, the vast majority of foragers instead exhibited substantial variation in both metrics across days. Specifically, most foragers specialized on collecting pollen or nectar on a given day, but individual foragers exhibited a surprising degree of flexibility in how specialized they were on any given day, and body size is fixed in adult insects.

In addition, our results suggest that at least for bumblebees, antennal sensory morphology does not directly account for short or long-term patterns of reward specialization. If lifetime reward preference reflected variation in response thresholds, as commonly presumed for honeybees^4^,^9^, and antennal sensory morphology in turn governed response thresholds^10^, short-term reward specialists (lifetime reward generalists) would be expected to exhibit intermediate numbers of pore plates. Yet we did not find that reward specialization was associated with pore plate number. Interestingly, we did find that workers with more pore plates made more pollen bouts over their lifetimes. Taken together with^10^, this result suggests that pore plates may mediate lifetime pollen foraging activity to some degree for eusocial pollinators generally.

Patterns of short-term specialization are one way in which colonies could meet shifting resource demands. However, fixed lifetime preferences could also allow colonies to meet demand for a given food type if lifetime specialists were able to rapidly and flexibly alter their foraging effort (number of bouts). For instance, if demand for pollen exceeded demand for nectar, lifetime specialists on nectar might decrease their foraging effort, while lifetime specialists on pollen might increase their foraging effort. Yet while we found that daily foraging effort varied nearly 40-fold across bees, an individual’s foraging effort did not change significantly over lifetime. In fact, approximately 17% of bees made 50% of the colony’s daily foraging bouts. Variation in bumblebee foraging effort was a continuous, though strongly skewed, distribution – similar to honeybees^44^. Our results thus indicate that while individual bumblebee foragers may exhibit tremendous flexibility in patterns of reward specialization over lifetime, foraging effort is not as plastic. Furthermore, greater lifetime foraging effort was associated with a greater number of pore plates (and thus larger body size). This result lends additional support to the hypothesis that larger bees with better sensory capabilities also contribute more to nutrient collection^21^,^22^,^38^.

Our results suggest a number of future directions. In this study we forced bees that collected both pollen and nectar to switch between foraging chambers. This design imposed a cost of switching between rewards, which could have contributed to the observed patterns of specialization. Future work should therefore directly investigate whether switching between collection of pollen and nectar is costly (as suggested for bees foraging from live flowers^41^) and whether imposing greater switching costs might cause a greater degree of short-term reward specialization. Switching costs may be greater under natural conditions, as distances between plants can be large. Likewise, the effect of differences in forager morphology on reward specialization may be more pronounced in the field. For instance, a longer proboscis permits use of flowers with deeper nectaries, and bees with larger wings can forage at a larger spatial scale^21^,^24^,^45^. At the same time, flowers of many plant species offer pollen and nectar simultaneously. Therefore we might expect foragers to exhibit less reward specialization over the course of a bout when foraging from these flowers, reflecting a reduction in switching costs. To our knowledge no studies have yet formerly quantified patterns of reward specialization under these conditions.

Furthermore, although honeybee-collected pollen is regularly used in studies of bee foraging behaviour (e.g., refs 20, 36 and 46) this reward is adulterated with debris and up to 60% sugars^25^ and is thus not a realistic substitute for floral pollen that wild bees must collect. While behavioural patterns in our study were robust, the use of honeybee pollen might be misleading, especially where the collection of pollen is concerned. For instance, adulteration with nectar may have reduced the putative role of pore plates in mediating pollen foraging behaviour in our study. Future work should thus have bees forage for live (unadulterated) floral pollen.

In closing, we report here a more complete accounting of lifetime patterns of foraging for alternative floral rewards than has been conducted to date. We found that while lifetime reward specialization in bumblebees is rare, short-term specialization on either pollen or nectar appears to be the norm. While reward specialists were not larger or more active foragers, we propose that patterns of short-term specialization in particular allow bumblebees colonies to flexibly meet shifts in pollen and nectar demand. Future work will be required to investigate what factors regulate short-term specialization and whether reward specialists at either lifetime or daily timescales also enhance colony performance, for instance, by making quicker bouts or carrying larger resource loads than generalists at either timescale. Moreover, we found enormous interindividual variation in foraging effort; a small number of foragers contributed disproportionately to foraging effort. Future studies should examine whether heightened foraging activity simply reflects a general heightened activity level (a ‘behavioural syndrome’^47^).

## Additional Information

**How to cite this article:** Russell, A. L. *et al*. Patterns of pollen and nectar foraging specialization by bumblebees over multiple timescales using RFID. *Sci. Rep.* **7**, 42448; doi: 10.1038/srep42448 (2017).

**Publisher's note:** Springer Nature remains neutral with regard to jurisdictional claims in published maps and institutional affiliations.

## Supplementary Material

Supplementary Information
