# Supplementary material for: Patterns of pollen and nectar foraging specialization by bumblebees over multiple timescales using RFID
Source: Sci Rep. 2017 Feb 9;7:42448. doi: 10.1038/srep42448 (PMC5299450; doi:10.1038/srep42448)
Supplement: Supplementary Information [file srep42448-s1.doc]

SUPPLEMENTARY MATERIALS

TITLE: Patterns of pollen and nectar foraging specialization by bumblebees over multiple timescales using RFID

Avery L. Russell^a*^, Sarah J. Morrison^b^, Eleni H. Moschonas^c^, and Daniel R. Papaj^c^

*Experimental foraging setup*

RFID readers in a pair were held 3cm apart using foam: readers with less separation fail to read transponders. To reduce the chance of readers failing to detect tagged bees, the floor of each reader’s tunnel (through which the bee traversed and was scanned) was slightly elevated to bring bees closer to the readers using custom built plastic pieces (LxWxH 8 x 2 x 1.1 mm) taped in place.

*RFID data processing software*

A foraging bout (or ‘bout’) was assigned using the following method. An ‘in’ (IN) event was scored as the detection of an RFID tag by the outermost reader (distal to the colony box), of the pair attached to each foraging chamber, followed by a detection of that same tag by the innermost reader (proximal to the colony box). Conversely, an ‘out’ (OUT) event was scored as a detection from the innermost reader followed by that from the outermost reader. We set a threshold time difference value between inner and outer reader detections (‘pairing threshold’) to score whether detections were paired (and therefore constituted a bee leaving or entering the nest as part of a foraging bout). If a bee took longer than 60 seconds to pass through the two readers, the two different reader detections were scored as unpaired (occasionally bees will stop between readers, leading to this problem).

A complete foraging bout consists of a bee generating an OUT event followed by an IN event, thus necessitating 4 detections in total (2 detections for each reader pair). The failure to record any of these 4 required detections disqualified the putative bout from further analysis. We scored foraging bouts over the lifetime of each bee. The first day of foraging for each bee were excluded when examining patterns of individual foraging behaviour to ensure our dataset only included days during which bees could forage throughout the day. The duration of a given foraging bout was calculated by determining the time elapsed between paired OUT and IN events (with the average time point of paired reader detections serving as start and end points for a foraging bout).

Each RFID tag can be read multiple times as a bee passes through a reader. We set a threshold time difference value for combining sequential same-tag detections (‘same bout threshold’). This threshold also affects the number of unpaired detections. For instance, if a bee switches from travelling into the colony to going out of the colony 15 seconds after the last detection from the same reader, a second bout was counted. If a bee took less than 15 seconds to make this manoeuvre, no second bout was counted. For a subset of the data (days 1-5) 96.5% of the 82609 sequential same-tag detections that occurred fell below this 15-second threshold. The mean same-tag detection interval was 4.16 ± 0.26 seconds; the median was 0.184 seconds.

Reader failure rate was estimated for 1 hour of observations for each of 5 consecutive days, involving 79 bouts. Zero of these events failed to be recorded by either reader, while 77 involved only one reader reporting a tag. Thus 3% of all interactions failed to report a foraging bout, resulting in a reader failure rate similar to that reported in other studies^S1,42^. We assumed reader failures occurred with the same frequency for each bee.

*No systematic asymmetry between forewings and between antennae*

We found little difference between forewings and between antennae for each bee and no systematic asymmetry (paired *t*-tests: pore plate number, *t*_68_ = 0.487, *P* = 0.628; forewing length, *t*_108_ = 0.124, *P* = 0.902; mean percent similarity ± SE: pore plate number, 93.65 ± 0.83, range = 219-505, *N* = 69; forewing length, 97.60 ± 0.30, *N* = 109).

*Model selection for data analysis*

To determine whether morphological characteristics were associated with behavioural patterns we first used multivariate multiple regression models (MMRs). MMRs were specified via the lm() function in R. We used multiple MMRs to retain power and eliminate errors; we therefore grouped variables in MMRs by similarity (lifetime variables grouped in one MMR, daily mean variables grouped in a separate MMR). With one MMR we examined effects on lifetime behaviour (‘total days foraged’, ‘lifetime bouts’, ‘lifetime nectar bouts’, ‘lifetime pollen bouts’, ‘lifetime nectar preference’). For this MMR we log transformed ‘lifetime bouts’, ‘lifetime nectar bouts’, ‘lifetime pollen bouts’ and thereby normalized the residuals. A second MMR was used to determine effects on mean daily behaviour (‘mean daily bouts’, ‘mean daily nectar bouts’, ‘mean daily pollen bouts’). For this second MMR we log transformed the response variables and thereby normalized the residuals. Morphological characteristics (‘forewing length’, ‘head -width’, ‘proboscis length’, ‘pore plate number’, ‘pore plate density’) were specified as independent variables, while behavioural characteristics were specified as response variables with the R function cbind(). To simplify these MMRs we applied backwards elimination via the mStep() function in the qtlmt package^S2^. For the first MMR all independent variables aside from ‘pore plate number’ were eliminated; for the second MMR all independent variables aside from ‘forewing length’ were eliminated. We subsequently report results of linear models (LMs) for each separate dependent variable, applying a conservative Bonferroni correction (α-value = 0.006).

**Table S1: Correlation (*r***) between morphological measures

|  | **Forewing length** | **Head width** | **Proboscis length** | **Pore plate number** | **Antennal length** | **7th ant. segment width** | **7th ant. segment length** | **Pore plate density** |
| --- | --- | --- | --- | --- | --- | --- | --- | --- |
| **Forewing length** |  | 0.90 | 0.84 | 0.76 | 0.92 | 0.85 | 0.82 | -0.20 |
| **Head width** | 0.90 |  | 0.74 | 0.66 | 0.97 | 0.89 | 0.88 | -0.35 |
| **Proboscis length** | 0.84 | 0.74 |  | 0.66 | 0.75 | 0.69 | 0.64 | -0.10 |
| **Pore plate number** | 0.76 | 0.66 | 0.66 |  | 0.73 | 0.70 | 0.65 | 0.33 |
| **Antennal length** | 0.92 | 0.97 | 0.75 | 0.73 |  | 0.92 | 0.91 | -0.30 |
| **7^th^ ant. seg. width** | 0.85 | 0.89 | 0.69 | 0.70 | 0.92 |  | 0.86 | -0.38 |
| **7^th^ ant seg. length** | 0.82 | 0.88 | 0.64 | 0.65 | 0.91 | 0.86 |  | -0.41 |
| **Pore plate density** | -0.20 | -0.35 | -0.10 | 0.33 | -0.30 | -0.38 | -0.41 |  |

All morphological measures, except for pore plate density, were strongly correlated with one another. Eight bees were discarded due to missing values in one or several morphological categories; *N* = 101 bees.

**Table S2: Correlation (*r***) between lifetime behavioural measures

|  | **Lifetime bouts** | **Lifetime nectar bouts** | **Lifetime pollen bouts** | **Total days foraged** | **Lifetime nectar preference** |
| --- | --- | --- | --- | --- | --- |
| **Lifetime bouts** |  | 0.97 | 0.75 | 0.59 | 0.28 |
| **Lifetime nectar bouts** | 0.97 |  | 0.56 | 0.47 | 0.38 |
| **Lifetime pollen bouts** | 0.75 | 0.56 |  | 0.69 | -0.1 |
| **Total days foraged** | 0.59 | 0.47 | 0.69 |  | 0.01 |
| **Lifetime nectar preference** | 0.28 | 0.38 | -0.1 | 0.01 |  |

All lifetime behavioural measures were strongly correlated with one another, except for lifetime nectar preference with total days foraged, or with lifetime pollen bouts. Eleven bees that did not make enough bouts to calculate lifetime nectar preference were discarded. *N* = 98 bees.

**Figure S1**


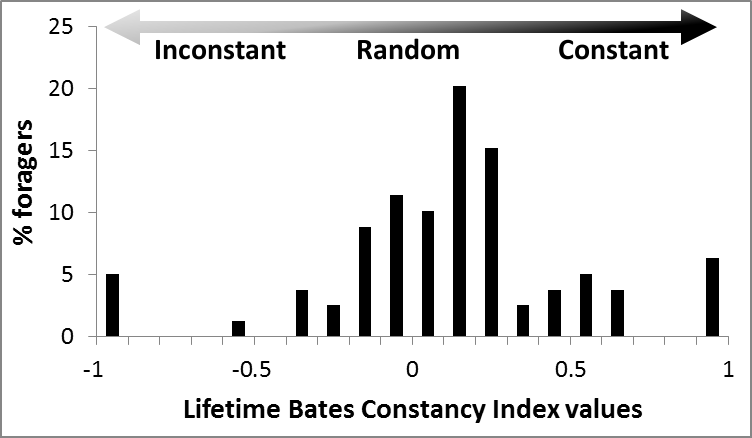


**Figure S1**. Bates Constancy Index (CI) for forager lifetime: bees that were more inconstant (systematically alternated between rewards) have values closer to -1; bees that made random transitions between rewards have values closer to 0; bees that foraged in runs for one or the other reward have values closer to 1; bin width = 0.1; mean Bates CI ± SE = 0.12 ± 0.05; *N* = 79 bees.

SUPPLEMENTARY REFERENCES

1. Molet, M., Chittka, L. & Raine, N. E. Potential application of the bumblebee foraging recruitment pheromone for commercial greenhouse pollination. *Apidologie* **40**, 608-616 (2009).
2. Cheng, R. Tools for mapping multiple complex traits. R package version 0.1-4 [cited 2016 Oct 8]. https://cran.r-project.org/web/packages/qtlmt/qtlmt.pdf (2015).
